# The Association between the Planetary Health Diet with a Regular Consumption of Breakfast and a Well-Balanced Diet: A Cross-Sectional Analysis Involving Japanese Male Engineering Students at a University in Hyogo Prefecture

**DOI:** 10.3390/ijerph21070858

**Published:** 2024-06-29

**Authors:** Etsuko Kibayashi, Makiko Nakade

**Affiliations:** 1 Department of Food and Nutrition, Sonoda Women’s University, Amagasaki 661-8520, Hyogo, Japan; 2 Department of Food Science and Nutrition, University of Hyogo, Himeji 670-0092, Hyogo, Japan; nakade@shse.u-hyogo.ac.jp; 3 Research Institute for Food and Nutritional Sciences, Himeji 670-0092, Hyogo, Japan

**Keywords:** Japanese male undergraduates, planetary health diet, eating breakfast, healthy diets, dietary quality, health behaviour

## Abstract

Few young Japanese adults regularly consume a well-balanced diet composed of staples, main courses, and sides. We hypothesised that adopting the recommended planetary health diet with regular breakfast consumption would promote well-balanced diets among young Japanese male undergraduates. This study aimed to examine the structural association between the planetary health diet with regular breakfast consumption and a well-balanced diet intake. This cross-sectional study included 142 male engineering undergraduates who completed a 2022 online questionnaire via Google Forms at a public university in Hyogo Prefecture. The planetary health diet comprises the consumption of the recommended eight items. A covariance structure analysis was performed in a hypothetical model with factors (regular breakfast consumption and eight items) potentially associated with the intake of a well-balanced diet at least twice daily. After excluding chicken dishes and nuts from the eight recommended items of the planetary health diet, goodness-of-fit became acceptable. Frequent consumption of the remaining six recommended items (fish, eggs, soybeans/soybean products, dairy foods, vegetables, and fruits) was significantly positively correlated with regular breakfast consumption and a significant positive path to a well-balanced diet. Among male university students, regular breakfast consumption and the planetary health diet may lead to a well-balanced diet.

## 1. Introduction

The food-based dietary guidelines issued in 2000 (partly revised in 2016) for the Japanese population [1] recommend well-balanced meals comprising staples (cereal grains), main courses (proteins), and sides (vegetables). Furthermore, the ‘Japanese Food Guide Spinning Top’ was devised in 2005. Mortality risk from cardiovascular [2,3] and cerebrovascular [3] diseases decreased in individuals who adhered to the sex- and age-recommended daily amounts of various food groups, as indicated in the 2005 Japanese Food Guide Spinning Top [4]. The Japanese government has set a goal to ensure that the proportion of Japanese people who consume meals comprising staple foods, main courses, and side dishes at least twice daily increases to at least 50% of the population by 2025. However, the percentage in 2020 remained low at 36.4% [5]. The Hyogo Nutrition and Diet Survey conducted in 2021 showed that among respondents aged ≥20 years, compared with males and females aged 60–69 years (38.9% and 47.3%, respectively), the proportion of those who consumed meals that comprised staples, main courses, and side dishes at least twice a day on ‘6 or 7 days per week’ was the lowest among males and females aged 20–29 years (26.5% and 25.8%, respectively), followed by males and females aged 30–39 years (27.4% and 32.7%, respectively); the proportion was the lowest among the younger generation [6].

Besides the problem of not consuming well-balanced diets, younger adults have another problem: skipping breakfast. Daily breakfast consumption is recommended by health and nutrition professionals and governments worldwide [7]. Consuming breakfast is associated with better dietary quality [8,9] and helps prevent stroke in Japan [10]. According to a survey from 28 countries, skipping breakfast was associated with inadequate fruit and vegetable intake, frequent soft drink intake, not avoiding fat and cholesterol, current binge drinking, less healthy lifestyles, mental problems, and poor academic performance [11]. It has also been reported that female sex, smoking, night eating syndrome, poor sleep quality, and being overweight/obese are associated with a higher likelihood of skipping breakfast [12]. Skipping breakfast decreased the HEI-2010 component scores for total fruit, whole fruit, whole grains, total dairy, and empty energy and increased the scores for total vegetables and refined grains [9]. However, decreasing breakfast consumption has been reported in many countries [13]. Similarly, Japan is not exempt from this behaviour; according to the 2017 National Health and Nutrition Survey [14], the percentage of individuals who skipped breakfast was the highest in males and females aged 20–29 years (30.6% and 23.6%, respectively). The Hyogo Nutrition and Diet Survey conducted in 2021 [6] showed that among the respondents aged ≥ 20 years, the proportion of those who ate breakfast ‘6 or 7 days per week’ was the lowest among males, compared with females, aged 20–29 years (47.0% vs. 64.9%). It has been suggested that not only unhealthy lifestyles and eating habits, but also low health awareness, are associated with the habit of skipping breakfast among Japanese university students [15].

The United Nations’ Sustainable Development Goals (SDG) [16] necessitate a transition to sustainable food systems, which involves major improvements in food production practices, a substantial shift toward mostly plant-based diets, and marked reductions in food loss and waste [17], to reduce environmental impacts and achieve a better food future. The EAT-Lancet Commission confirmed the need for a shift toward the planetary health diet [18]; accordingly, the Sustainable Healthy Diets Guiding Principles [19] were published by the WHO in 2019. Moreover, food-based dietary guidelines implemented with the assistance of the Food and Agriculture Organization of the United Nations (FAO) incorporate the need for sustainability. Thus, the planetary health diet is recommended to achieve the SDGs worldwide. A recent survey in Japan [20] showed an SDG awareness rate of 86.0%, which had increased by more than 30% and approximately six times from that in the fourth and first surveys conducted in January 2021 and February 2018, respectively. When stratified by sex and age, SDG awareness was higher among teenage males (94.6%) and those in their 30s (91.8%). Therefore, relatively young adults are expected to be slightly more interested in planetary environment-related issues rather than health-related issues. As breakfast consumption has been associated with better dietary quality [8,9], we hypothesised that combined adherence to the recommended planetary health diet and regular breakfast consumption might promote the adoption of a well-balanced diet among young Japanese males. This theory indicates the need to examine the associations of planetary health diet consumption with regular breakfast consumption and the intake of a well-balanced diet to promote a healthy diet among young adults. However, few studies have examined the association between recommending the planetary health diet and the consumption of a well-balanced diet. In addition, few studies have comprehensively examined the relationship between regular breakfast consumption and adherence to the planetary health diet and a well-balanced diet.

In this study, we aimed to comprehensively examine the associations between regular breakfast consumption and adherence to the planetary health diet and well-balanced diets among Japanese male university students. The planetary health diet encompasses foods recommended to achieve the SDGs, and this study assessed the association of the planetary health diet and eating breakfast with consuming a well-balanced diet in this demographic. Recommending the planetary health diet [18,21] should aid in promoting a healthy diet and ameliorating prevalent issues [6,14], such as poorly balanced diets and irregular breakfast habits among young adults.

## 2. Materials and Methods

### 2.1. Study Design and Participants

This study was conducted at a public university in Hyogo Prefecture, Japan. The population of this study consisted of engineering students aged 18–24 years (the total number of the enrolled engineering students was 1624: 1397 males and 227 females). Because the proportion of males aged 20–29 years who ate breakfast ‘6 or 7 days per week’ was lower than that of females [6], we targeted the engineering students, many of whom were male, in this study. In this study, we recruited students only through a university portal site, and by use of flyers and posters. We asked the university students to complete a questionnaire survey through the notice board of the university portal site and flyers with a QR code for Google Forms. The response period was from 7 November to 10 December 2022. Posters with QR codes were also displayed at conspicuous locations across the university. In total, 222 students responded to the questionnaire. Among them, female students (*n* = 37) and those who had missing values were excluded, leaving 142 eligible male students (age: 20.0 ± 1.3 years) who were selected (80.6% valid response rate) for analysis by non-probability.

This study was conducted in accordance with the tenets of the Declaration of Helsinki, and all procedures were approved by the Ethics Committee of the University of Hyogo (number 297: this study was approved on 14 November 2022). Information including the purpose of the study, the right to refuse to participate, and the security of personal information was provided to all participants before their participation in this study, and survey respondents were considered to have consented to participate in this study.

### 2.2. Measures

The questionnaire was created by the first author by referencing the items of previous studies and national/prefectural nutrition surveys. The second author evaluated the questionnaire, and then, all authors discussed and modified the items of the questionnaire. The questionnaire used in this study included items pertaining to the respondents’ characteristics, including age, frequency of eating a well-balanced diet (6 or 7 days/4 or 5 days/2 or 3 days/≤1 day/not at all), living arrangement (living alone or with family), regular exercise (exercise for ≥30 min) (3 times per week for ≥1 year/2 times per week for ≥1 year/2 times per week and <1 year continuously/once per week/little or no exercise/no exercise at all), frequency of eating breakfast (6 or 7 days/4 or 5 days/2 or 3 days/≤1 day/not at all), attempting to consume an environmentally friendly diet (working on it already (>6 months)/working on it already (<6 months)/intend to start soon (within 1 month)/intend to start soon (within 6 months)/interested but not working on it/not interested), and self-reported height and body weight. Body mass index (BMI) was calculated as body weight (kg)/height (m)^2^. A well-balanced diet was defined as the regular consumption of meals composed of staples (cereal grains), main courses (proteins), and side dishes (vegetables) at least twice daily.

Food constituting the planetary health diet was defined according to the Japanese food groups and diet with reference to a summary report of the EAT-Lancet Commission [21], excluding grains, which form a majority of the staple foods consumed in Japanese diets [22], i.e., the consumption of three ‘limited intake’ items (beef, pork, and potato dishes) and eight ‘optional or emphasised food’ items (chicken dishes, fish dishes, egg dishes, soybeans/soybean products, nuts, dairy foods, vegetable-based dishes, and fruit). In the Japanese food groups, among legumes, soybeans/soybean products were selected as one of the emphasised items because they are the most consumed legumes [23]. The students were asked to choose only one option for the frequency of each item and were assigned scores as follows: 7 = at least twice daily, 6 = once daily, 5 = 5 or 6 times/week, 4 = 3 or 4 times/week, 3 = 1 or 2 times/week, 2 = 1–3 times/month, and 1 = not at all for each item. The reliability of the 11-item (limited intake/optional or emphasised food) scale of the foods constituting the planetary health diet was confirmed in this analysis (142 participants) with a Cronbach’s alpha coefficient of 0.8, which is acceptable for internal consistency. Item analysis revealed that when items were deleted, Cronbach’s alphas for all but “chicken dishes” (0.806) fell between 0.769 and 0.793. The adjusted item total correlations were slightly lower for “chicken dishes” at 0.216 but ranged from 0.375 to 0.636 for the others.

### 2.3. Data Analysis

The mean values for age by well-balanced diet (eating a well-balanced diet regularly: ≥4 days/week or not eating a well-balanced diet regularly: ≤3 days/week or less) were compared by Student’s *t*-test, and proportions (BMI, living arrangement) by well-balanced diet regularity were compared by the chi-square test. The variables of regular exercise, frequency of eating breakfast, and attempt to consume an environmentally friendly diet were analysed as an interval scale. Welch’s *t*-test was used to compare the above-specified items by well-balanced diet regularity. Then, for the association between a well-balanced diet and the planetary health diet, the dependent variable was the well-balanced diet, and the independent variable was the planetary health diet. Binomial logistic regression analysis (forced entry method) was conducted with age and living arrangement (living alone or with family) as adjustment factors, and odds ratios and 95% confidence intervals were calculated. The dependent variable was 1 for eating a well-balanced diet regularly and 0 (standard) for not eating a well-balanced diet regularly. The independent variable was consumption of each item of the planetary health diet “more than 3 times a week (only nuts: ≥2 times/week)”, set to 1, and “less than 2 times a week (only nuts: ≤3 times/month)”, set to 0 (standard).

**Figure 1:**
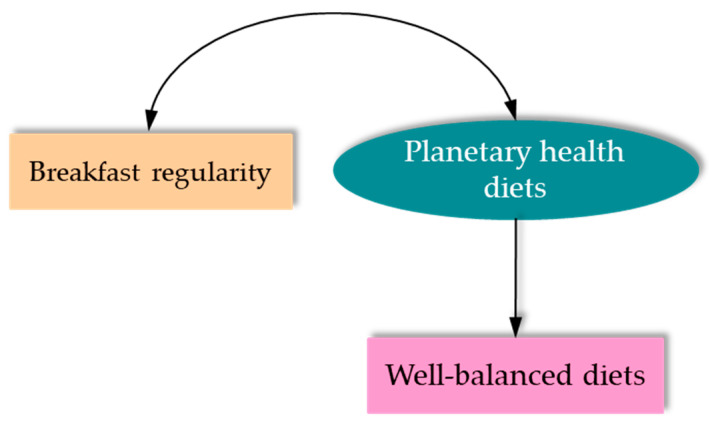
The initial hypothetical model associating well-balanced diets with the consumption of items from the planetary health diet (the eight items [optional or emphasised]) and breakfast regularity. The bidirectional arc arrow shows an association, and the straight arrows indicate significant paths.

We then developed an initial hypothetical model (Figure 1) associating a well-balanced diet with the consumption of items from the planetary health diet (the eight items [optional or emphasised]) and breakfast regularity as an effect modifier. We conducted a covariance structure analysis to validate the hypothetical model. In the analysis, path direction, standardised estimates, the coefficient of determination, the goodness-of-fit index (GFI), the adjusted GFI (AGFI), the comparative fit index (CFI), root mean square error of approximation (RMSEA), and Akaike’s information criterion (AIC) were estimated. The model was modified repeatedly by deleting non-significant paths until the best possible model fit was achieved. We determined that the goodness of fit of the model was better when the GFI, AGFI, and CFI indices were ≥0.9, RMSEA was ≤0.05, and AIC was lower than that of the other models. The sample size was calculated using RMSEA for the null hypothesis, ε0 ≤ 0.1, and RMSEA for the alternative hypothesis, ε1 = 0.01, with the power of the not-close fit test = 0.8, the model degrees of freedom = 19, and a significance level of 5%, resulting in the minimum sample size being 117 [24]. Statistical analyses were performed using SPSS version 26 (IBM Japan, Ltd., Tokyo, Japan, 2019), with statistical significance set at *p* < 0.05.

## 3. Results

Table 1 shows the characteristics of the participants and the results of intergroup comparison stratified by regular consumption of a well-balanced diet. Comparisons of well-balanced diet regularity showed a significant intergroup difference (*p* = 0.016) in age (mean age 19.8 years and 20.3 years, respectively) among participants with and without regular well-balanced diet consumption. Among participants who consumed a well-balanced diet regularly, those living with family accounted for the highest percentage (88.4%). In contrast, among participants who did not eat a well-balanced diet regularly, 50.7% lived alone, with a significant intergroup difference (*p* < 0.001). Comparisons of well-balanced diet regularity showed no intergroup difference in the frequency of attempts to eat a environmentally friendly diet; however, there was a significant intergroup difference (*p* < 0.001) in the frequency of eating breakfast 6 or 7 days/week between participants with and without regular well-balanced diet consumption (72.5% and 43.8%, respectively).

The results of the binomial logistic regression analysis on the association between the consumption of a well-balanced diet (more than 4 days a week/less than 3 days a week) and the planetary health diet (frequent consumption of limited intake/optional or emphasised foods) are shown in Table 2. The odds ratios of those eating fish dishes more than three times a week (odds ratio [95% confidence interval]: 2.76 [1.19, 6.39]), eating soybeans/soybean products more than three times a week (2.81 [1.42, 5.55]), eating vegetable-based dishes more than three times a week (3.51 [1.61, 7.62]), and eating fruit more than three times a week (3.34 [1.60, 7.00]) were significantly higher among those who consumed a well-balanced diet regularly than among those who did not eat a well-balanced diet regularly. After adjusting for age and living arrangement (living alone or with family), those eating fish dishes more than three times a week (3.01 [1.15, 7.88]), soybeans/soybean products more than three times a week (2.56 [1.20, 5.49]), and fruit more than three times a week (2.29 [1.03, 5.12]) were significantly more likely to consume a well-balanced diet.

**Figure 2:**
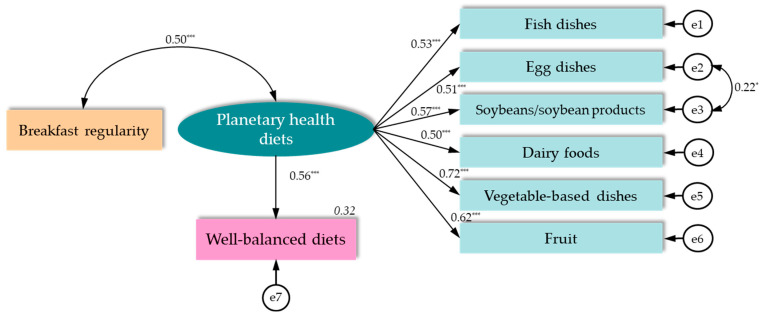
The association of a well-balanced diet with the consumption of items from the planetary health diet (frequent consumption of the six optional or emphasised items) and breakfast regularity among Japanese male undergraduates (*n* = 142). The numbers in regular font in the path diagram are standardised estimates (next to the straight arrows) and correlation coefficients (above the bidirectional arc arrow). The numbers in italics are R^2^ values (coefficients of determination). *: *p* < 0.05, **: *p* < 0.01, and ***: *p* < 0.001. Goodness of fit: χ^2^ = 24.586, *df* = 19 (*p* = 0.175), GFI = 0.962, AGFI = 0.927, CFI = 0.975, RMSEA = 0.046, AIC = 58.586.

In the initial hypothetical model (Figure 1), the results did not show acceptable goodness of fit (χ^2^ = 69.419, *df* = 34, GFI = 0.915, AGFI = 0.863, CFI = 0.873, RMSEA = 0.086, and AIC = 111.419). By excluding chicken dishes and nuts from the eight recommended items of the planetary health diet, an acceptable goodness of fit was obtained (χ^2^ = 24.586, *df* = 19, GFI = 0.962, AGFI = 0.927, CFI = 0.975, RMSEA = 0.046, AIC = 58.586) (Figure 2). Frequent consumption of the remaining six items of the planetary health diet, namely fish, eggs, soybeans/soybean products, dairy foods, vegetables, and fruits, had a significant positive correlation (0.50, *p* < 0.001) with breakfast regularity and a significant positive path (standardised estimate 0.56, *p* < 0.001) to transition to a well-balanced diet.

## 4. Discussion

In this study, we investigated whether recommending regular breakfast habits and the planetary health diet [18,21], including chicken, fish, eggs, soybeans/soybean products, nuts, dairy foods, vegetables, and fruits, might help ameliorate dietary issues [6,14] such as poorly balanced diets among young Japanese adults. Accordingly, a structural analysis of the covariance of the hypothesised model was performed using planetary health diet consumption, regular breakfast consumption, and well-balanced diet intake among engineering students at a university in Hyogo Prefecture.

After excluding chicken and nuts, the final hypothetical model of this study comprised six of the eight recommended items of the planetary health diet, namely, fish, eggs, soybeans/soybean products, dairy foods, vegetables, and fruits. In previous studies of dietary transition in Japan [25], the frequency of chicken in breakfasts published in Japanese culinary journals is extremely low compared to that of eggs and dairy products; therefore, the intake of chicken dishes was excluded from the final model. The analysis also excluded the intake of nuts, potentially due to their infrequent consumption. Nuts are not commonly consumed in Japanese dietary habits.

Among the G20 countries, Japan has relatively low per capita food-related greenhouse gas emissions [26,27]. Furthermore, adherence to a well-balanced diet, characterised by a combination of staple foods, main courses, and side dishes, as outlined in the 2000 Japanese food-based dietary guidelines, is currently recommended. The origin of Japanese cuisine is attributed to ‘*honzen ryori*’ (*honzen* cuisine), which was used to entertain guests of the samurai families in the Muromachi period (AD 1336–1573), and its basic form is ‘*ichijyu sansai*’ (one soup and three dishes: vinegared, simmered, and grilled), in addition to rice and savoury dishes [28,29,30]. Throughout its extensive history, Japanese food culture has permeated the existing dietary habits of the Japanese people, which includes eating ‘*okazu*’ (the main course and side dishes) with rice—the staple food—as the main ingredient. Dairy products were not a part of the common people’s diet until after World War II. The Ministry of Health and Welfare (now the Ministry of Health, Labour and Welfare) established a Ministerial Ordinance on Ingredient Standards for Dairy Products in 1951. This is possibly why the current daily intake of Japanese people remains at a low average of 110.7 g [23]. However, in this study, dairy foods were included among the six recommended items of the planetary health diet. The percentage of adults (age ≥ 20 years) who ate rice for breakfast daily (17.7% in the 2016 Hyogo Dietary Survey) [31] was lower than that 13 years ago (24.8% in 2003). In contrast, the highest percentage of breakfast content in the 2016 Hyogo Dietary Survey [31] comprised staple foods (91.9%), whereas dairy foods accounted for 47.6%, which was higher than the 37.5% and 36.7% contents of main and side dishes, respectively. This may be due to the intake of dairy foods, along with bread and non-rice cereals, as a staple for breakfast. Therefore, because they are regularly consumed at breakfast, dairy foods have been retained as one of the six items in the planetary health diet.

For more than 20 years, the National Health and Nutrition Survey in Japan has indicated that the lower consumption of fish (50.8 g vs. 59.2 g), soybeans/soybean products (46.2 g vs. 63.4 g), vegetables (222.6 g vs. 268.6 g), and fruits (46.9 g vs. 70.6 g) per day among young adults than among middle-aged and older adults (age groups 20s vs. 50s, respectively) constitutes a problem [23]. In this study, dietary issues in young adults were related to four of the six recommended items in the planetary health diet. Therefore, improving the intake of these items in combination with regular breakfast consumption may lead to a healthy and well-balanced diet.

The importance of breakfast has been reported in various countries, including resulting in higher dietary quality among Australian adults [32] and European adolescents [33] who consumed breakfast and highlighting the contribution of breakfast to overall dietary quality among American [9] and Filipinos [34] adults. Previous studies from 28 countries and from America also suggested skipping breakfast led to unhealthy food intake [9,11]. Among participants who ate a well-balanced diet regularly, 75.4% of them ate breakfast 6–7 days/week (Table 1). Our results support the previous findings and suggest that eating breakfast may lead to the consumption of a well-balanced diet. The intake of foods associated with the planetary health diet may vary slightly in each country. Nonetheless, the combination of the recommended planetary health diet and regular breakfast consumption may be an effective strategy to improve diet quality not only in Japan but also in other countries worldwide.

The strengths of the present study include the examination of dietary issues such as adherence to a balanced diet and skipping breakfast among young Japanese individuals, which may be ameliorated through an interventional approach that incorporates a focus on the planetary health diet to achieve the SDGs. Importantly, our study also integrated the findings that foods that were identified as problematic through low consumption among male engineering students at a university in Hyogo Prefecture were nearly identical to the foods recommended in the planetary health diet.

The limitations of this study include the fact that the participants were male engineering students at a university in Hyogo Prefecture. Therefore, they are not representative of the 18- to 24-year-old Japanese population. The low response rate was also a serious limitation. In this study, we recruited students only through a university portal site, flyers, and posters. The response rate might be higher if the data were collected in the classroom before or after the class. The difference between the students who responded to the survey and those who did not is unclear; however, they may have been more interested in diet and followed dietary recommendations better than others. Further research involving females and other department participants of different ages should be conducted. Moreover, because this was a cross-sectional study, we were unable to ascertain a causal relationship between regular breakfast consumption-related changes in adherence to the recommendations of the planetary health diet and habituation to a well-balanced diet. An additional study of the effects of a well-balanced diet through interventional research that promotes regular breakfast consumption according to the recommendations of the planetary health diet for young adults will be helpful in this regard. Validation of the questionnaire on the consumption of each item of the planetary health diet in this study also warrants further research, including its reproducibility in participants’ responses. Furthermore, the R^2^ values (coefficients of determination) for well-balanced diets in this study model are low and can only explain the 32% well-balanced diet trend. Because the lifestyle and eating habit factors as well as the low health awareness associated with eating breakfast among Japanese university students have been reported [15], future studies including such factors may clarify a more comprehensive relationship with following a well-balanced diet. Although there are limitations as described above, few studies have examined the comprehensive relationship between regular breakfast intake and adherence to the planetary health diet or a balanced diet, as in this study, and these findings should be useful in the future to promote the consumption of a well-balanced diet among young Japanese males.

In summary, we examined the structural associations between planetary health diet consumption and regular breakfast consumption and well-balanced diet intake in young males. In male university students, the association of regular breakfast consumption with adherence to the planetary health diet, which includes foods recommended to achieve the SDGs, may lead to the consumption of a well-balanced diet.

## 5. Conclusions

In this study, we have suggested that recommending regular breakfast habits and the planetary health diet, which helps meet the SDGs set by the United Nations, might help ameliorate dietary issues such as poorly balanced diets among young Japanese male undergraduates.

## Figures and Tables

**Table 1:** Characteristics of participants and intergroup comparison stratified by well-balanced diet regularity (*n* = 142).

Characteristics	Total	Eating a Well-Balanced Diet Regularly ^1^	Not Eating a Well-Balanced Diet Regularly ^1^	*p*
*n*	142 (100)	69 (48.6)	73 (51.4)	
Age ^2^	20.0 ± 1.33	19.8 ± 1.25	20.3 ± 1.35	0.016
BMI ^2^, kg/m^2^				
<18.5	21 (14.8)	9 (13.0)	12 (16.4)	0.61
≥18.5 and <25	113 (79.6)	55 (79.7)	58 (79.5)	
≥25	8 (5.6)	5 (7.2)	3 (4.1)	
Living arrangement ^3^				
Living alone	45 (31.7)	8 (11.6)	37 (50.7)	<0.001
Living with family	97 (68.3)	61 (88.4)	36 (49.3)	
Regular exercise (exercise for ≥30 min) ^4^				
3 times/week for ≥1 year	28 (19.7)	13 (18.8)	15 (20.5)	0.90
2 times/week for ≥1 year	19 (13.4)	11 (15.9)	8 (11.0)	
2 times/week, <1 year continuously	22 (15.5)	9 (13.0)	13 (17.8)	
Once per week	26 (18.3)	15 (21.7)	11 (15.1)	
Little or no exercise	40 (28.2)	17 (24.6)	23 (31.5)	
No exercise at all	7 (4.9)	4 (5.8)	3 (4.1)	
Eating behaviours				
Frequency of eating breakfast ^4^				
6 or 7 days/week	82 (57.7)	50 (72.5)	32 (43.8)	<0.001
4 or 5 days/week	19 (13.4)	10 (14.5)	9 (12.3)	
2 or 3 days/week	17 (12.0)	3 (4.3)	14 (19.2)	
≤1 day/week	7 (4.9)	2 (2.9)	5 (6.8)	
Not at all	17 (12.0)	4 (5.8)	13 (17.8)	
Attempt to consume environmentally friendly diet ^4^				
Working on it already (>6 months)	14 (9.9)	8 (11.6)	6 (8.2)	0.53
Working on it already (<6 months)	14 (9.9)	8 (11.6)	6 (8.2)	
Intend to start soon (within 1 month)	8 (5.6)	4 (5.8)	4 (5.5)	
Intend to start soon (within 6 months)	22 (15.5)	8 (11.6)	14 (19.2)	
Interested, but not working on it	53 (37.3)	26 (37.7)	27 (37.0)	
Not interested	31 (21.8)	15 (21.7)	16 (21.9)	

The data are expressed as mean ± SE or *n* (%). ^1^ Eating a well-balanced diet regularly: ≥4 days/week; not eating a well-balanced diet regularly: ≤3 days/week. ^2^ Student’s *t*-test was used. ^3^ Ratios were compared by two independent sample chi-squared tests. ^4^ Welch’s *t*-test was conducted, as the data were analysed as interval scales (6 = 3 times/week for at least 1 year; working on it already (>6 months). 5 = 2 times/week for at least 1 year; 6 or 7 days/week; working on it already (<6 months). 4 = 2 times/week, <1 year continuously; 4 or 5 days/week; intend to start soon (within 1 month). 3 = once per week; 2 or 3 days/week; intend to start soon (within 6 months). 2 = little or no exercise; 1 day/week or less; interested but not working on it. 1 = no exercise at all; not at all; not interested).

**Table 2:** The association between a well-balanced diet (more than 4 days a week/less than 3 days a week) and the planetary health diet (frequent consumption of limited intake/optional or emphasised food) (*n* = 142).

Characteristics	Total	Without Adjustment OR [95% CI]	*p*	With Adjustment ^1^ OR [95% CI]	*p*
*n*	142 (100)				
Beef dishes ^2^					
At least twice daily	1 (0.7)	1.09 [0.55, 2.17]	0.80	0.69 [0.32, 1.49]	0.34
Once daily	3 (2.1)				
5 or 6 times/week	7 (4.9)				
3 or 4 times/week	39 (27.5)				
1 or 2 times/week	53 (37.3)	1		1	
1–3 times/month	34 (23.9)				
Not at all	5 (3.5)				
Pork dishes ^2^					
At least twice daily	1 (0.7)	1.80 [0.89, 3.60]	0.10	1.58 [0.74, 3.39]	0.24
Once daily	3 (2.1)				
5 or 6 times/week	6 (4.2)				
3 or 4 times/week	40 (28.2)				
1 or 2 times/week	76 (53.5)	1		1	
1–3 times/month	12 (8.5)				
Not at all	4 (2.8)				
Potato dishes ^2^					
At least twice daily	1 (0.7)	1.96 [0.96, 4.03]	0.066	1.63 [0.73, 3.61]	0.23
Once daily	3 (2.1)				
5 or 6 times/week	11 (7.7)				
3 or 4 times/week	30 (21.1)				
1 or 2 times/week	55 (38.7)	1		1	
1–3 times/month	34 (23.9)				
Not at all	8 (5.6)				
Chicken dishes ^3^					
At least twice daily	4 (2.8)	1.11 [0.58, 2.15]	0.75	1.42 [0.67, 2.98]	0.36
Once daily	5 (3.5)				
5 or 6 times/week	14 (9.9)				
3 or 4 times/week	45 (31.7)				
1 or 2 times/week	59 (41.5)	1		1	
1–3 times/month	13 (9.2)				
Not at all	2 (1.4)				
Fish dishes ^3^					
At least twice daily	1 (0.7)	2.76 [1.19, 6.39]	0.018	3.01 [1.15, 7.88]	0.025
Once daily	2 (1.4)				
5 or 6 times/week	2 (1.4)				
3 or 4 times/week	26 (18.3)				
1 or 2 times/week	72 (50.7)	1		1	
1–3 times/month	30 (21.1)				
Not at all	9 (6.3)				
Egg dishes ^3^					
At least twice daily	4 (2.8)	1.55 [0.76, 3.14]	0.23	1.30 [0.59, 2.88]	0.52
Once daily	22 (15.5)				
5 or 6 times/week	27 (19.0)				
3 or 4 times/week	43 (30.3)				
1 or 2 times/week	35 (24.6)	1		1	
1–3 times/month	10 (7.0)				
Not at all	1 (0.7)				
Soybeans/soybean products ^3^					
At least twice daily	3 (2.1)	2.81 [1.42, 5.55]	0.003	2.56 [1.20, 5.49]	0.015
Once daily	13 (9.2)				
5 or 6 times/week	16 (11.3)				
3 or 4 times/week	36 (25.4)				
1 or 2 times/week	43 (30.3)	1		1	
1–3 times/month	25 (17.6)				
Not at all	6 (4.2)				
Nuts ^3^					
At least twice daily	1 (0.7)	1.35 [0.63, 2.85]	0.44	1.05 [0.46, 2.42]	0.90
Once daily	5 (3.5)				
5 or 6 times/week	2 (1.4)				
3 or 4 times/week	9 (6.3)				
1 or 2 times/week	20 (14.1)				
1–3 times/month	38 (26.8)	1		1	
Not at all	67 (47.2)				
Dairy foods ^3^					
At least twice daily	9 (6.3)	1.52 [0.78, 2.98]	0.22	1.01 [0.47, 2.15]	0.99
Once daily	38 (26.8)				
5 or 6 times/week	14 (9.9)				
3 or 4 times/week	20 (14.1)				
1 or 2 times/week	32 (22.5)	1		1	
1–3 times/month	19 (13.4)				
Not at all	10 (7.0)				
Vegetable-based dishes ^3^					
At least twice daily	14 (9.9)	3.51 [1.61, 7.62]	0.002	2.36 [0.99, 5.62]	0.052
Once daily	28 (19.7)				
5 or 6 times/week	20 (14.1)				
3 or 4 times/week	37 (26.1)				
1 or 2 times/week	33 (23.2)	1		1	
1–3 times/month	7 (4.9)				
Not at all	3 (2.1)				
Fruit ^3^					
At least twice daily	2 (1.4)	3.34 [1.60, 7.00]	0.001	2.29 [1.03, 5.12]	0.043
Once daily	18 (12.7)				
5 or 6 times/week	8 (5.6)				
3 or 4 times/week	19 (13.4)				
1 or 2 times/week	36 (25.4)	1		1	
1–3 times/month	43 (30.3)				
Not at all	16 (11.3)				

The data are expressed as *n* (%) or OR [95% CI: confidence interval]. Binomial logistic regression analysis (forced entry method) was used. The dependent variable was 1 for eating a well-balanced diet regularly and 0 (standard) for not eating well-balanced diet regularly, and the independent variable was the consumption of each item of the planetary health diet, with “more than 3 times a week (only nuts: ≥2 times/week)” set to 1, and “less than 2 times a week (only nuts: ≤3 times/month)” set to 0 (standard). ^1^ Adjustment factors were employed with age and living arrangement (living alone or with family). ^2^ A limited intake of food in the planetary health diet. ^3^ Optional or emphasised food in the planetary health diet.

## Data Availability

The data in this study are available from the corresponding authors upon request. The data are not available to the public for confidentiality reasons.
